# Second trimester abortion as a cause of maternal death: a case report

**DOI:** 10.11604/pamj.2015.22.261.7208

**Published:** 2015-11-19

**Authors:** Sümeyra Nergiz Avcioglu, Sündüz Özlem Altinkaya, Mert Küçük, Emre Zafer, Selda Demircan Sezer, Hasan Yüksel

**Affiliations:** 1 Department of Gynecology and Obstetrics, Adnan Menderes University, School of Medicine, Aydin, Turkey; 2 Department of Gynecology and Obstetrics, Mugla Sitki Koçman University, School of Medicine, Mugla, Turkey

**Keywords:** Second trimester, spontaneous abortion, maternal death

## Abstract

Each year, an estimated 529 000 maternal deaths occur worldwide. In literature, it is known that maternal mortality can occur during pregnancy, peripartum and also in postpartum period. Although very rare, maternal deaths may occur after spontaneous abortion. In present case, 37 year old G5P4 (Caesarean Section) women was admitted to Adnan Menderes University, Obstetrics and Gynecology clinic with diagnosis of missed abortion at 18 weeks’ gestation. She had been hospitalized in the public maternity hospital for five days due to abortus incipience and prolapse of amnion membranes but had no contractions. Fetal heart beats ceased at the second day of hospitalization. Medically induced abortion was recommended but not accepted by the patient. At the fifth day of hospitalization, she was referred to our clinic due to deterioration of general health condition, low blood pressure and tachycardia. In emergency department, it was determined that she was not oriented, had confusion, had blood pressure of 49/25 mmHg and tachycardia. In ultrasonographic examination, 18 week in utero ex fetus was determined and there was free fluid in abdominopelvic cavity. The free fluid was suspected to be amniotic fluid due to rupture of uterus. Laparotomy was performed, no uterine rupture, hematoma or atony was observed. However during laparotomy, a very bad smelling odor, might be due to septicemia, was felt in the operation room. Cardiac arrest occurred during that operation. In autopsy report, it was concluded that maternal death was because of remaining of inutero ex fetus for a long time. In conclusion, although very rare, maternal deaths after spontaneous abortion may occur. Because spontaneous abortion is a common outcome of pregnancy, continued careful, strict monitoring and immediate treatment of especially second trimester spontaneous abortion is recommended to prevent related, disappointing, unexpected maternal deaths.

## Introduction

Maternal mortality is one of the important maternal health parameters. Since 1990, worldwide maternal death rates have declined by 47% although the incidence is still high in developing countries. The global maternal mortality rate is about 210/100000 births, while in developing countries it is 240/100000 compared to 14/100000 in developed countries [1]. At the international and national level, accurate identification and timely recording of maternal deaths are needed. Death of a woman during pregnancy or within 42 days of termination of pregnancy is defined as maternal death. In that terminology, termination of pregnancy is irrespective of the duration and site of the pregnancy and from any cause related to or aggravated by the pregnancy or its management [2]. There have been variable causes of maternal mortality. Hemorrhage is the leading cause of maternal deaths in Africa (33.9%) and Asia (30.8%) however hypertensive disorders were responsible for 25% of maternal mortality in Latin America and Caribbean [3]. Maternal death related to abortion is still determined in high incidences in underdeveloped and developing countries like Ghana, Afghanistan, Tanzania, Mexico, and Nigeria due to unsafe abortion and due to restriction of legal abortion by legislation [4]. It is observed about 13% of all pregnancies, including unsafe abortions [5]. However maternal mortality rate in developed countries was only 0.7 per 100000 abortions [6]. In present case, a maternal death due to disseminated intravascular coagulation (DIC) in a spontaneous abortion will be discussed.

## Patient and observation

In present case, 37 year old G5P4 (Caesarean Section) women was admitted to Adnan Menderes University, Obstetrics and Gynecology clinic with diagnosis of missed abortion at 18 weeks’ gestation. She had been hospitalized in a public maternity hospital for five days due to abortus incipience and prolapse of amnion membranes but had no contractions. There was no history of rupture of amnion membranes. Fetal heart beats (FHBs) were determined positive during the first three days of hospitalization in the public maternity hospital. However, FHB ceased at the fourth day. Medically induced abortion was recommended but was not accepted by patient. Patient preferred and wished expectant management. During the follow up at maternity hospital, infection markers were all normal and there was no fever. At the fifth day, she was referred to our clinic due to deterioration of general health condition, low blood pressure and tachycardia. In emergency department, it was determined that she was not oriented, had confusion, had blood pressure of 49/25 mmHg, tachycardia (145 beats/min) and body temperature was 36.6°C. She had hyperventilation and oxygen saturation was 88%. First of all, bolus hydration with isotonic solutions and oxygen treatment was performed. In complete blood count she had hemoconcentration (Hemotocrit: 44%). In ultrasonographic examination, 18 weeks in utero ex fetus was determined and there was free fluid in abdominopelvic cavity. The free fluid was suspected to be amniotic fluid due to rupture of uterus. So, laparotomy was performed due to suspicion of uterine rupture and clinical deterioration. Through the vertical incision, ex fetus was taken out. However during that procedure, a very bad smelling odor, which might be due to septicemia, was felt in the operation room. But there was no uterine rupture, hematoma, atony. Only little amount of serous fluid was determined in abdominal cavity. We thought that this fluid was due to extravasation of intercellular and intravascular fluid. Despite aggressive hydration therapy including blood products, by central venous catheterization, she had persistant bradycardia and hypotension. Vasopressin (0.03 units/min) was given to increase mean blood pressure to at least 60 mm Hg. Epinephrine was added as second drug. Dobutamine as an inotropic was also given. During operation, cardiac arrest occurred. Despite all resuscitation efforts, patient was accepted as exitus after half an hour. Autopsy was performed and in autopsy report, it was concluded that maternal death might be due to disseminated intravascular coagulation (DIC) because of remaining of ex fetus in uterus for a long time.

## Discussion

There have been different records for incidence of maternal mortality due to abortion. Mortality rates are still high after an abortion or stillbirth [7]. According to World Health Organization (WHO), abortion accounts for 7.9% of maternal deaths worldwide [8]. In Turkey, abortion accounts for only 1-2% of maternal deaths. Little is known about maternal deaths after spontaneous abortion. Risk factors for these deaths were age over 29 years, being black and unmarried status. All over the world, most deaths (58%) occurred between 12 and 19 weeks′ gestation. The risk of death was much higher for abortion in the second trimester compared with first trimester [9]. In the present case report, patient was older than 29 years, had missed abortion at second trimester, which were risk factors for abortion. In contrast to risk factors for abortion, she was white and married.

In a research, it was determined that infection (48%), hemorrhage (21%), embolism (11%), and other causes (20%) may lead to mortality due to spontaneous abortion [9]. Again in another study investigating deaths due to spontaneous abortions in United States during 1981-1991, found that, deaths mostly occurred due to infection (59%) and hemorrhage (18%) [10]. In autopsy, it was concluded that maternal death had occurred due to DIC because of persistence of inutero ex fetus for a long time. Besides, in autopsy report, there has been no clue about embolism in any organ especially in lung and brain. On the other hand there was no history or clinical finding of hemorrhage either before hospitalization or during hospitalization at the public maternity hospital. Infection was the only suspected explanation of the reason for DIC. An awful odor felt in operation room during operation, supported that issue. Sampling of uterine cavity and fluid in intra-abdominal cavity for culture was performed but it was negative. It was very interesting that infection markers were all normal and patient had no fever recorded during follow up in maternity hospital. However laboratory findings including culture results may be negative in septic shock [11]. Similar to present case report, in literature, mostly DIC was an associated condition among half of those deaths for which it was not the primary cause of death [10].

In the present case, induced abortion was recommended after fetal demise but patient wished expectant management. In literature there have been conflicting data about expectant management of missed abortion. In a study it was determined that expectant management for two weeks from day of diagnosis was successful in 58% of cases and it was a safe option as none of the patients on expectant/medical management needed strong analgesia/antibiotics or blood transfusion in that study [12]. However the data in that literature might not be suitable for all patients as in our case.

## Conclusion

In conclusion, although very rare, maternal deaths after spontaneous abortion may occur. Because spontaneous abortion is a common outcome of pregnancy, continued careful, strict monitoring and immediate treatment of especially second trimester spontaneous abortion is recommended to prevent related, disappointing, unexpected maternal deaths.
